# Sunitinib-related high-grade proteinuria and allograft dysfunction in a kidney recipient: a rare case report

**DOI:** 10.1186/s12882-022-02789-5

**Published:** 2022-04-18

**Authors:** Hsu-Cheng Ko, Huai-Pao Lee, Jiann-Der Wu, Tsung-Liang Ma, Cheng-Huang Shen, Chang-Te Lin, Ming-Chin Cheng, Yeong-Chin Jou

**Affiliations:** 1 Department of Urology, Ditmanson Medical Foundation, Chia-Yi Christian Hospital, Chia-Yi, Taiwan; 2 Department of Pathology and Laboratory Medicine, Kaohsiung Veterans General Hospital, Kaohsiung, Taiwan; 3 Department of Pathology and Laboratory Medicine, Ditmanson Medical Foundation, Chia-Yi Christian Hospital, Chia-Yi, Taiwan; 4 Department of Nephrology, Ditmanson Medical Foundation, Chia-Yi Christian Hospital, Chia-Yi, Taiwan; 5 Department of Health and Nutrition Biotechnology, Asian University, Taichung, Taiwan

**Keywords:** Sunitinib, Nephrotic syndrome, Renal allograft dysfunction, Case report

## Abstract

**Background:**

Sunitinib-induced high-grade proteinuria and irreversible renal allograft dysfunction are rare conditions. Here, we present a patient who had received renal allograft and later developed metastatic clear cell renal cell carcinoma(cc-mRCC), for which he was prescribed sunitinib. High-grade proteinuria, hypoalbuminemia, peripheral edema and renal allograft dysfunction (manifesting as an increase in the serum creatinine concentration) occurred 5 months after sunitinib prescription.

**Case presentation:**

The patient was a 58-year-old male who had end-stage renal disease with regular hemodialysis through arteriovenous fistula for 17 years since 1998 and received a renal allograft from a deceased kidney donor in 2015. Unfortunately, in 2019, the patient developed cc-mRCC originating from the left native kidney. We suggested a needle biopsy on left native kidney or radical left nephrectomy, but the patient refused. Sunitinib was prescribed. Follow-up urine analysis showed proteinuria (500 mg/dL) 2 weeks after sunitinib prescription. He was hospitalized 5 months later because of body weight gain, decreased urine output, pitting edema of both lower extremities, and shortness of breath. The image studies showed progression in his cc-mRCC. His serum creatinine level and spot urine protein at admission increased to 4.26 mg/dL and 300 mg/dL, respectively. He agreed on a biopsy for the renal allograft and the pathology studies showed focal segmental glomerulosclerosis, acute interstitial nephritis, and acute tubular injury. Based on the time sequence of clinical presentations with the laboratory and pathological findings, sunitinib-induced renal allograft dysfunction secondary to high-grade proteinuria was most likely. Despite of discontinuation of sunitinib and increased dose of everolimus, renal impairment progressed. Thus, he had to receive hemodialysis starting 2 week after hospitalization. Unfortunately, the patient died of advanced metastasis despite of aggressive medical treatments 3 weeks after admission.

**Conclusion:**

This case report is a reminder that renal allograft dysfunction can happen secondary to proteinuria after taking sunitinib. Hence, clinicians must regularly check renal function and urine protein for renal allograft recipients. Monitoring and modifying drug prescription, especially sunitinib, is necessary if persistent proteinuria accompanied by deteriorating serum creatinine level occurs. Renal biopsy may be considered if more evidence is required to make a differential diagnosis.

**Supplementary Information:**

The online version contains supplementary material available at 10.1186/s12882-022-02789-5.

## Background

Sunitinib acts as one of the first-line tyrosine kinase inhibitor (TKI) in the treatment of metastatic clear cell renal cell carcinoma (cc-mRCC). Potential adverse events of sunitinib have been reported (e.g., renal dysfunction and nephrotic syndrome) [1, 2]. However, the use of sunitinib as well as its renal complications among renal allograft recipients is rarely reported. Here, we present a patient who developed cc-mRCC after renal transplantation and later presented renal allograft dysfunction secondary to proteinuria after sunitinib prescription.

## Case presentation

We present a 58-year-old male who had end-stage renal disease and underwent regular hemodialysis through arteriovenous fistula for 17 years. He received a renal allograft from a deceased kidney donor in February 2015. Methylprednisolone, tacrolimus, and mycophenolic acid were prescribed after transplantation.

Just after renal transplantation, we performed the biopsy of renal allograft. The result of histology showed no appearance of sclerosed glomerulus. Besides, there was no glomerulus found under the field of electron microscope. His serum creatinine (SCr) level was maintained between 1.52 and 2.08 mg/dL for the following years.

Unfortunately, he suffered from body weight loss, chronic cough, and sting-like pain on the left chest wall 4 years after transplantation. Non-enhanced abdominal and chest computed tomography (CT) revealed several nodular lesions at bilateral lung fields, multiple para-aortic enlarged lymph nodes, and a 3.2–cm mass in the left native kidney. CT-guided nodule biopsy of the right lung was smoothly performed. The immunohistochemical staining showed clusters of neoplastic cells expressing PAX-8, cytokeratin 7, and CD10 and vimentin with negative expression of cytokeratin 20, TTF-1, P40, GATA-3, and CD56. Therefore, cc-mRCC was diagnosed. Left renal biopsy or radical nephrectomy was suggested but the patient refused. He was then transferred to medical oncology where sunitinib 50 mg/day with dosing schedule of 3 weeks on and 1 week off initially. Due to intolerable toxicities to sunitinib, the dose was adjusted to 25 mg/day. Mycofenolic acid also shifted to everolimus 1 mg per day after the diagnosis of malignancy.

**Fig. 1:**
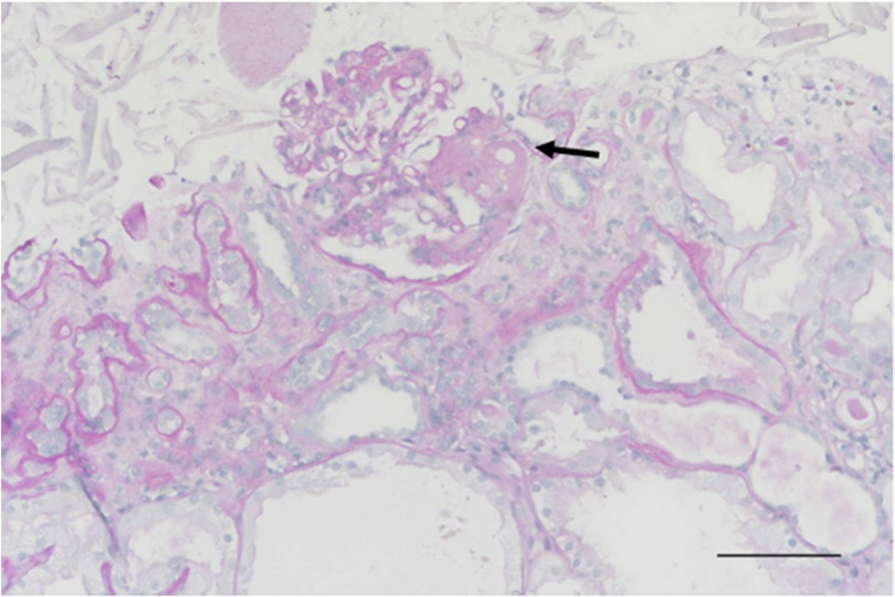
Focal segmental glomerulosclerosis with hyalinosis (arrow) (PAS staining, × 200, scale bar = 100 μm). For other supporting fields of cells, please refer to Supplementary information

**Fig. 2:**
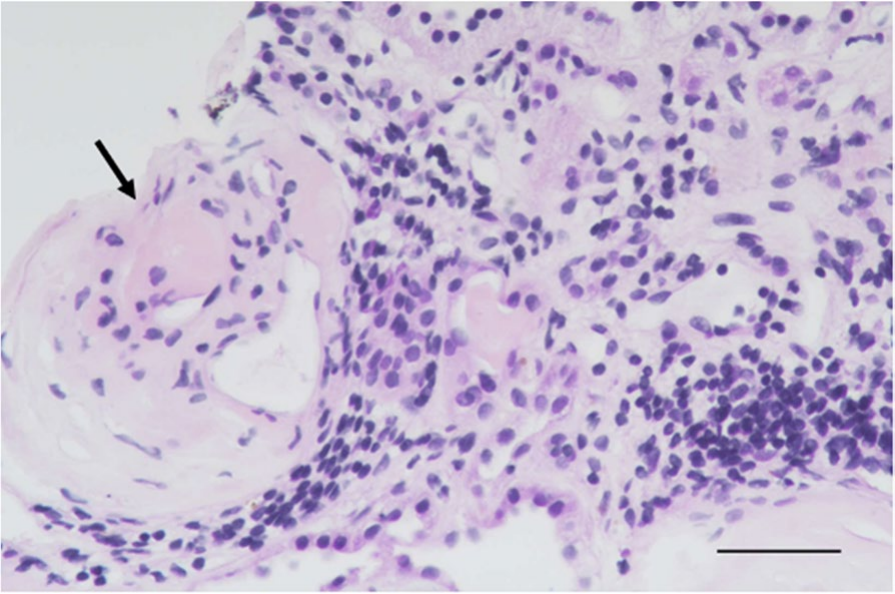
Cortical interstitial inflammation, characterized by lymphocytes and plasma cells, and a globally sclerosed glomerulus (arrow) in this field (H&E staining, × 400, scale bar = 50 μm). For other supporting fields of cells, please refer to Supplementary information

His urine analysis revealed proteinuria (500 mg/dL) after 0.5 month of sunitinib prescription. He was brought to our hospital due to body weight gain (from 49 kg before admission to 57 kg upon admission), decreased urine output, pitting edema at the bilateral lower extremities, and shortness of breath 5 months after the diagnosis of cc-mRCC. He was then admitted to the nephrology department under the impression of renal allograft dysfunction with oliguria. During hospitalization, image studies revealed progression of his cc-mRCC. SCr and spot urine protein increased to 4.26 mg/dL and 300 mg/dL, respectively. Serum albumin level was 2.8 g/dL. Consequently, a renal allograft biopsy was performed. The pathological findings of the biopsy revealed the following: A total of 11 glomeruli were identified on light microscopy, including three with global sclerosis and six with segmental sclerosis. The segmentally sclerosed glomeruli showed hyalinosis and focal podocyte hypertrophy (Fig. 1). Approximately 20% of the cortex showed tubular atrophy, 20% showed interstitial fibrosis, and 26% showed inflammation. The inflammatory cells were composed of lymphocytes and plasma cells, with focal lymphoid inflammatory cell aggregation in the interstitium (Fig. 2). There was also acute tubular injury featuring tubular lumen dilatation, tubular epithelial cell swelling, attenuated brush border of proximal tubules, and flattening and possibly loss of tubular epithelial cells. Tubulitis was not identified. Immunohistochemical staining was negative for C4d, polyomavirus, and cytomegalovirus. The immunohistochemical staining of glomeruli showed negative for IgG and IgA; faint focal segment in IgM, C1q, C3, kappa and lambda light chains. The type of equipment (microscopes/objective lenses, cameras, detectors, filter model and batch number) and acquisition software were shown as following. Nikon Eclipse E600 was the used microscope. Nikon Plan 20X and Nikon Plan 40X were used objective lenses in Figs. 1 and 2, respectively. Nikon DS-Ri2 was the used camera and detector. The filter model is NCB11. The batch number is 764099. NIS-Elements D 4.40 was the used acquisition software. We also performed the electron microscope. However, the specimen sent for electron microscope showed no renal tissue but only unremarkable fibroadipose tissue. After considering the time sequence of clinical symptoms and signs, increase in SCr and urine protein levels, drug consumption, and pathological results, we determined that sunitinib-induced renal allograft dysfunction secondary to high-grade proteinuria were impressed.

**Fig. 3:**
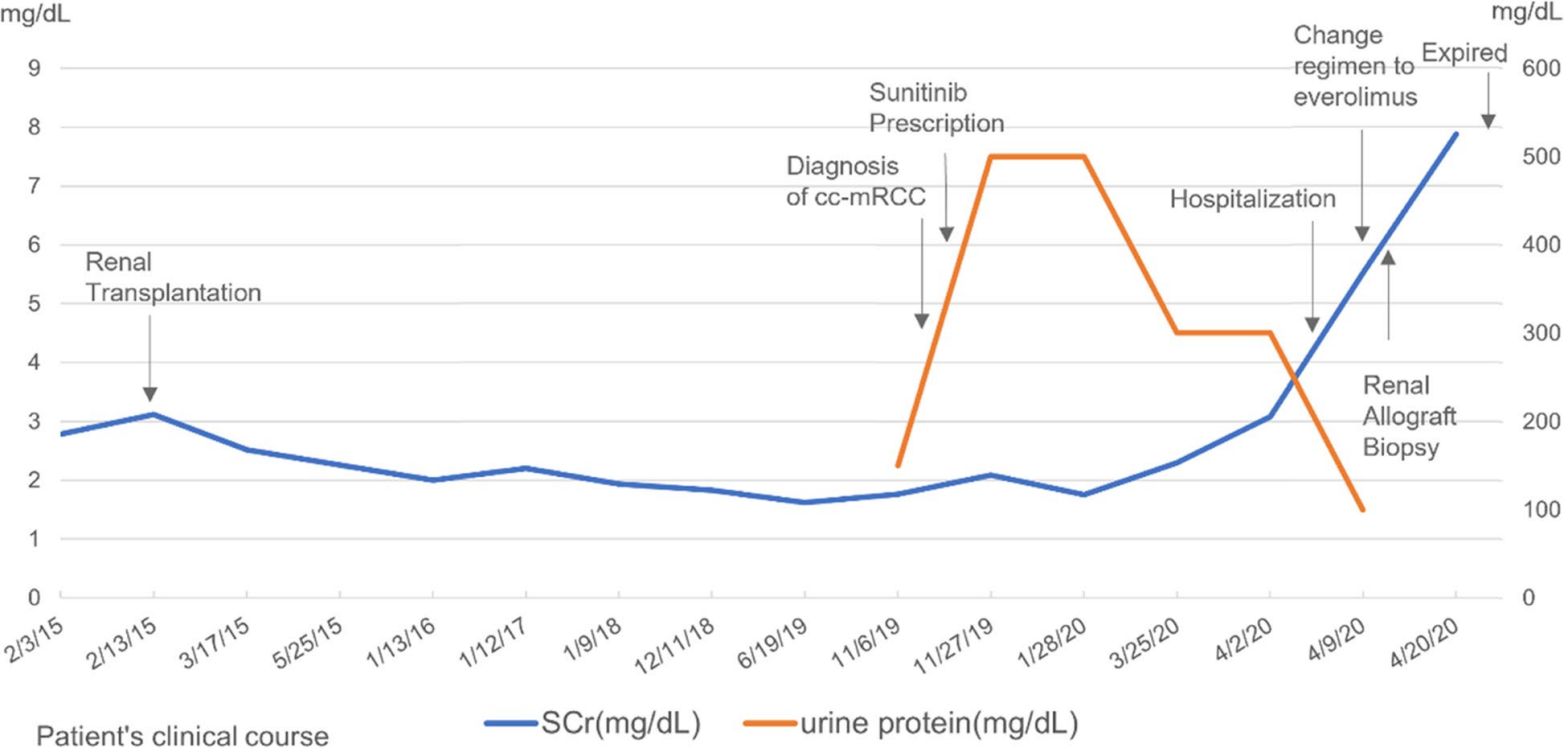
Patient’s clinical course, serum creatinine level and urine protein level

Sunitinib was discontinued and everolimus was increased from 1 mg per day to 5 mg per day for treatment of RCC as well as immunosuppression. However, graft function kept on deteriorating despite of medication switch. Thus, he had to receive hemodialysis through arteriovenous fistula thrice per week 1 week after hospitalization. During hospitalization, he also suffered from septicemia due to Pseudomonal infection. With prescription of cefepime, the condition of infection was under control. The immunosuppressive regimen was adjusted with only methylprednisolone (4 mg daily) left. However, he died 3 weeks after hospitalization because of metastatic complications and his family refused autopsy. His clinical course, serum creatinine level and urine protein level are summarized in the Fig. 3.

## Discussion and conclusions

Renal allograft recipients have a higher risk of developing RCC than the general population [3]. Among these recipients, the estimated incidence of de novo RCC in the native kidney and renal allograft were 0.7 and 0.2%, respectively [4]. Moreover, RCC metastasis occurs in approximately 30% of cases [5]. Hence, metastatic RCC is not uncommon in renal allograft recipients without timely detection. The first-line systemic treatments for cc-mRCC include sunitinib [6] and pazopanib [7], for those who cannot receive or tolerate immune checkpoint inhibitors according to the 2021 updated European Association of Urology guidelines on Renal Cell Carcinoma [8].

Sunitinib is an oral multi-targeted TKI of the vascular endothelial growth factor (VEGF) signaling pathway. The common side effects of sunitinib include hypertension, skin toxicity, and gastrointestinal disturbance. In the licensing trial of sunitinib, the elevation of SCr is more common in patients receiving sunitinib than in those receiving placebo [6]. Furthermore, a multicenter, randomized, phase-3 trial shares similar result regarding serum creatinine elevation [9]. However, in both sunitinib licensing and phase-3 trials, no report of nephrotic syndrome or acute renal failure exists. Several patients receiving sunitinib experienced a preeclampsia-like syndrome characterized by reversible hypertension, proteinuria, and edema [10, 11]. The incidences of proteinuria and renal insufficiency related to sunitinib were 18.9 and 7.7%, respectively, in a retrospective study from Korea. In most cases, renal function was preserved without deterioration after sunitinib discontinuation [12].

The pathology of renal biopsy can offer clinicians some evidence on renal damage associated with sunitinib aside from clinical symptoms of sunitinib-induced renal dysfunction. In the French Reins sous traitement Anti-VEGF Registre study, the renal biopsy of patients who were previously treated with sunitinib and developed nephrotic syndrome later showed FSGS, glomerular thrombotic microangiopathy, and acute tubular necrosis [11]. In another study, the pathology of renal biopsy shares similar results. Moreover, AIN is also a common finding [13]. The current case showed FSGS, AIN, and ATI in the renal allograft biopsy. The VEGF plays an important role in normal function and repair of glomerular endothelial cell [14]. Anti-VEGF therapy causes VEGF at low free level, which may induce endothelial dysfunction and podocyte dysregulation and lead to associated symptoms such as hypertension and proteinuria [15]. However, the clear pathophysiology of sunitinib related nephrotic syndrome and allograft dysfunction needs more studies to investigate.

Everolimus is a mTOR inhibitor with anti-tumor effect. Although several studies showed everolimus is inferior to sunitinib in treatment for RCC, everolimus was used as second line therapy [16], or in combination with VEGF inhibitors [17, 18]. Our patient with renal transplantation was treated with immunosuppressants including low dose everolimus after diagnosis of RCC. After progression of RCC refractory to sunitinib therapy, it was a reasonable way to increase dose of everolimus 1 mg per day to anti-cancer level with mildly reduced dose of 5 mg per day considering his progressive renal failure. Studies in renal transplantation recipients also showed lower incidence of malignancy after conversion from calcineurin inhibitors to mTOR inhibitors [19] and the safety of the conversion was confirmed even after diagnosis of post-transplant malignancy [20]. Although proteinuria is a common side effect of mTOR inhibitors, with possible mechanisms including reduced tubular protein reabsorption, podocyte dysregulation, and focal segmental glomerulosclerosis (FSGS) [21], it is not associated with acute interstitial nephritis (AIN). Besides FSGS, the pathologic examination of our patient’s graft kidney also showed AIN, more likely sunitinib-related.

This case is a reminder that sunitinib administration in kidney transplantation patients may result in an impairment of allograft dysfunction and severe proteinuria. Thus, clinicians must regularly check the renal function and urine analysis of renal allograft recipients. Moreover, medical prescription should be re-evaluated, especially for sunitinib, if persistent proteinuria accompanied with deteriorating serum creatinine level occurs.

There are some limitations in this case report, such as the failure to perform a biopsy on native kidney because the patient refused to receive further intervention then, and lack of demonstration of original disease in kidney biopsy before start of hemodialysis because he was at end stage renal disease upon his first visit at our nephrology department. We cannot totally exclude the diagnosis of de novo or recurrent FSGS in the case.

## Supplementary Information

**Additional file 1.**

## Data Availability

All the data relevant to this report are included in the manuscript.

## Abbreviations

TKI: Tyrosine kinase inhibitor
SCr: Serum creatinine
cc-mRCC: Metastatic clear cell renal cell carcinoma
CT: Computed tomography
PAX-8: Paired box gene 8
TTF-1: Thyroid transcription factor 1
GATA-3: GATA binding protein 3 to DNA sequence [A/T] GATA[A/G]
FSGS: Focal segmental glomerulosclerosis
AIN: Acute interstitial nephritis
ATI: Acute tubular injury
C4d: Complement component 4d
VEFG: Vascular endothelial growth factor
VEGFR: Vascular endothelial growth factor receptor
mTOR: Mammalian target of rapamycin
