# Supplementary material for: Sunitinib-related high-grade proteinuria and allograft dysfunction in a kidney recipient: a rare case report
Source: BMC Nephrol. 2022 Apr 18;23:150. doi: 10.1186/s12882-022-02789-5 (PMC9014636; doi:10.1186/s12882-022-02789-5)

Focal segmental glomerulosclerosis with hyalinosis (arrow) (PAS staining, ×200)


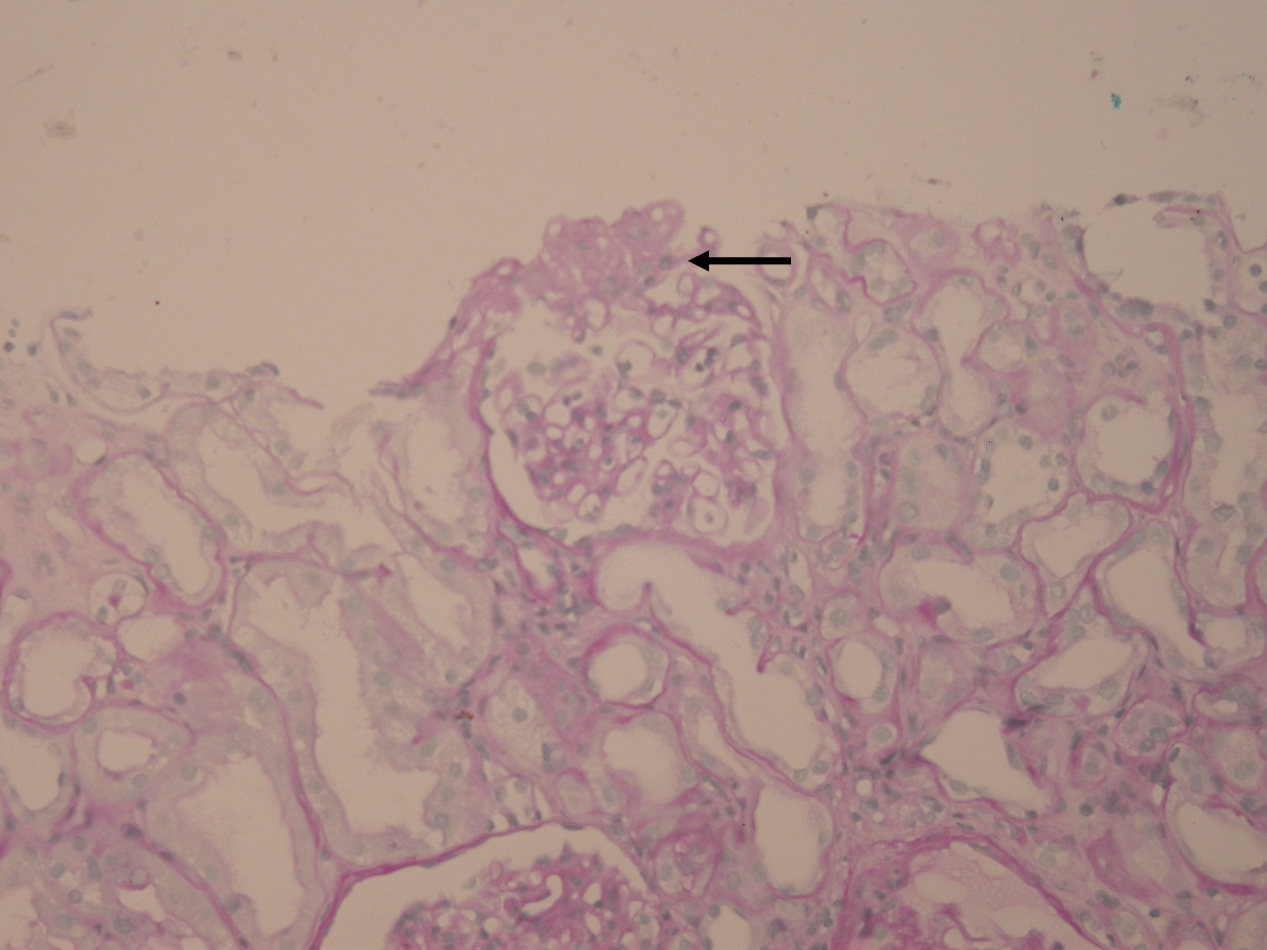


Focal segmental glomerulosclerosis with hyalinosis (arrow) (PAS staining, ×200)


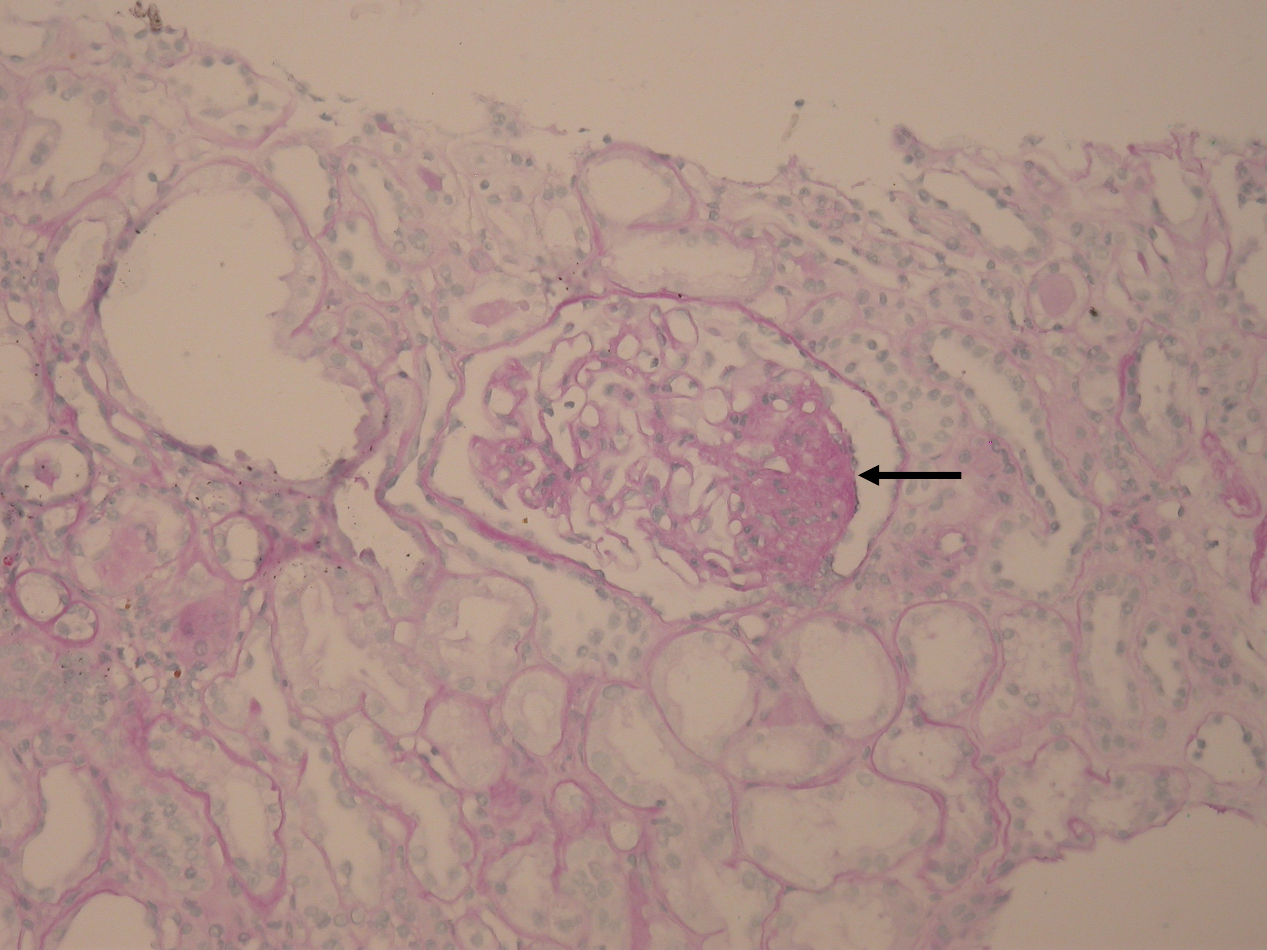


Cortical interstitial inflammation, characterized by lymphocytes and plasma cells, and focal segmental glomerulosclerosis (H&E staining, ×400)


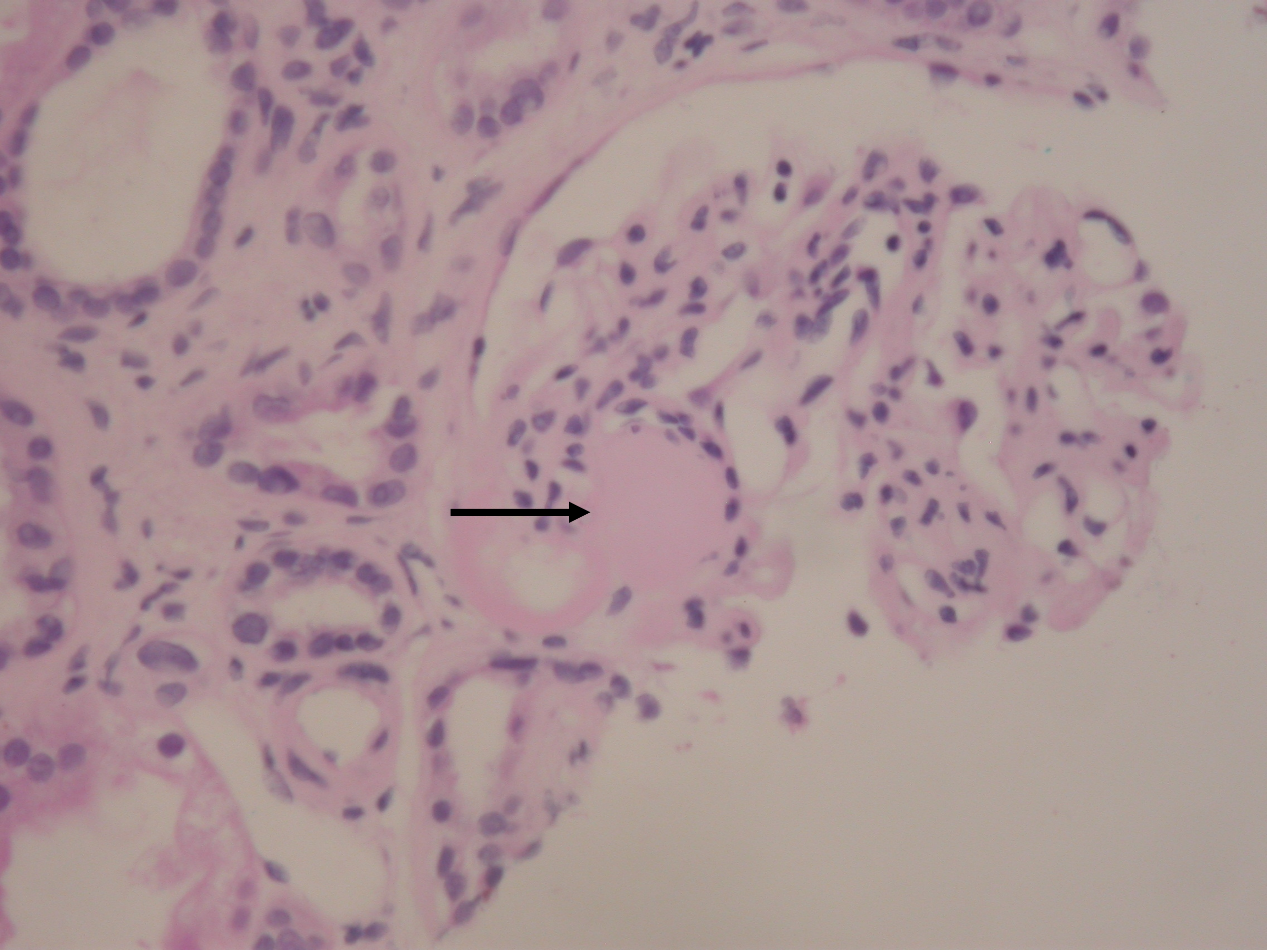


Cortical interstitial inflammation, characterized by lymphocytes and plasma cells, and focal segmental glomerulosclerosis (H&E staining, ×400)
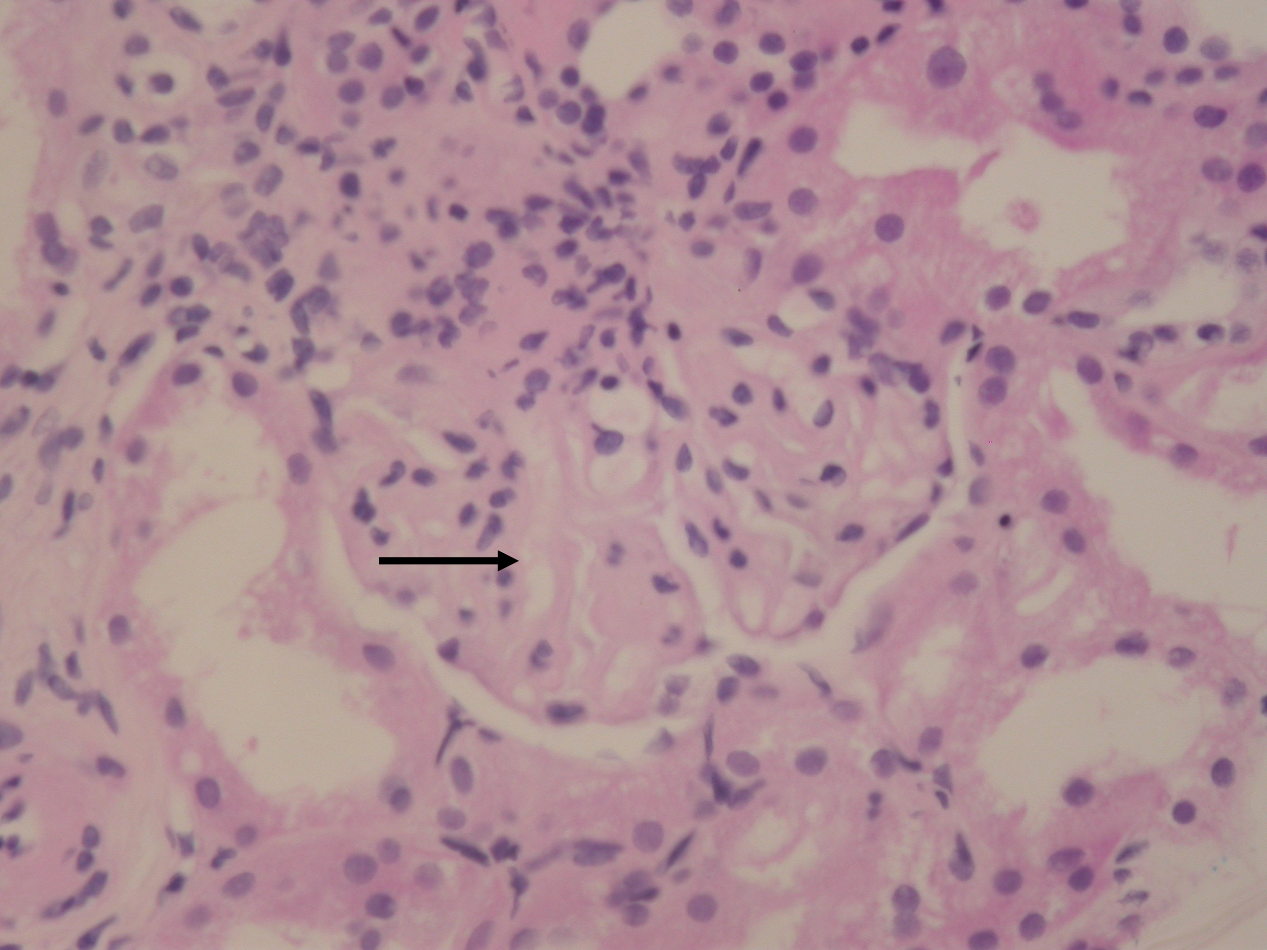


Cortical interstitial inflammation, characterized by lymphocytes and plasma cells, and focal segmental glomerulosclerosis (H&E staining, ×400)


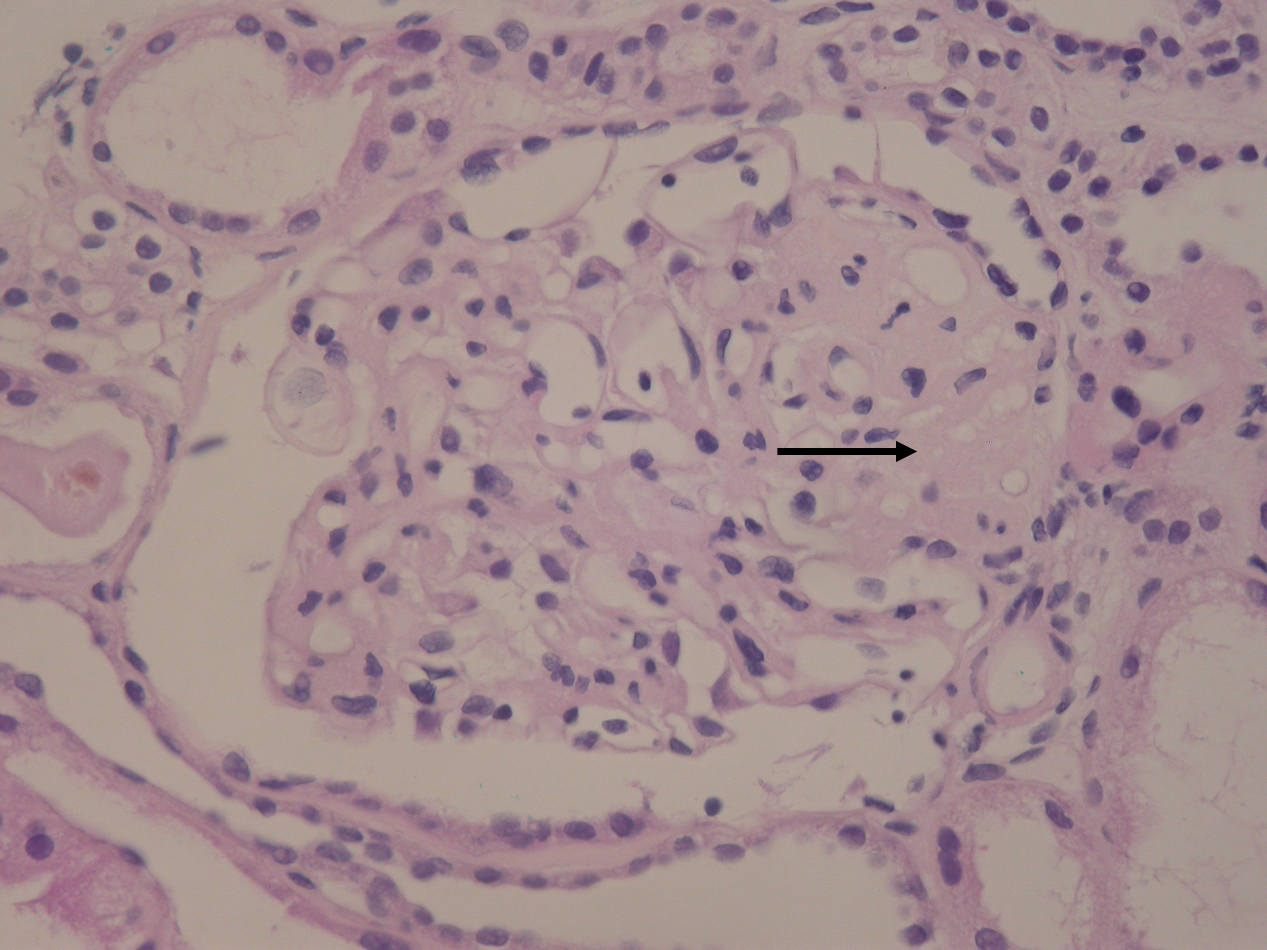

Supplement: Supplementary file 1 — Additional file 1. [file 12882_2022_2789_MOESM1_ESM.docx]
